# Exploring Marine Cyanobacteria for Lead Compounds of Pharmaceutical Importance

**DOI:** 10.1100/2012/179782

**Published:** 2012-04-01

**Authors:** Bushra Uzair, Sobia Tabassum, Madiha Rasheed, Saima Firdous Rehman

**Affiliations:** Department of Bioinformatics and Biotechnology, International Islamic University Islamabad, Sector H-10, 44000 Islamabad, Pakistan

## Abstract

The Ocean, which is called the “mother of origin of life,” is also the source of structurally unique natural products that are mainly accumulated in living organisms. Cyanobacteria are photosynthetic prokaryotes used as food by humans. They are excellent source of vitamins and proteins vital for life. Several of these compounds show pharmacological activities and are helpful for the invention and discovery of bioactive compounds, primarily for deadly diseases like cancer, acquired immunodeficiency syndrome (AIDS), arthritis, and so forth, while other compounds have been developed as analgesics or to treat inflammation, and so forth. They produce a large variety of bioactive compounds, including substances with anticancer and antiviral activity, UV protectants, specific inhibitors of enzymes, and potent hepatotoxins and neurotoxins. Many cyanobacteria produce compounds with potent biological activities. This paper aims to showcase the structural diversity of marine cyanobacterial secondary metabolites with a comprehensive coverage of alkaloids and other applications of cyanobacteria.

## 1. Introduction

**Figure 1:**
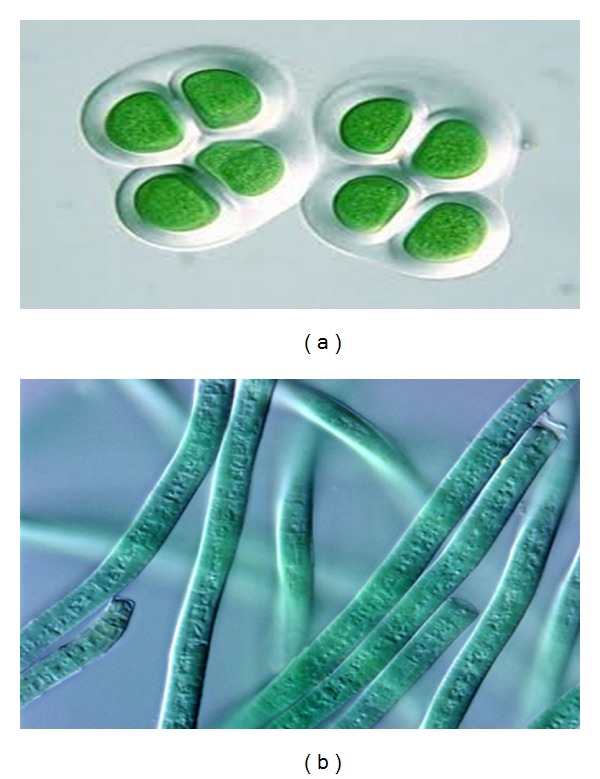
Nomarski differential interference contrasts photomicrographs of sheathed cyanobacterial strains. (a) *Chroococcus sp.* (1000x); (b) *Phormidium sp.* (1000x).

Cyanobacteria is a phylum of bacteria that obtain their energy through photosynthesis. The name “cyanobacteria" comes from the color of the bacteria. Cyanobacteria are a major and phylogenetically coherent group of Gram-negative prokaryotes possessing the unifying property of performing oxygenic plantlike photosynthesis with autotrophy as their dominant mode of nutrition [1]. However, in spite of their typically aerobic photosynthetic nature, some of the cyanobacterial species can grow in the dark on organic substrates [2] and others under anaerobic conditions with sulfide as electron donor for photosynthesis [3]. Certain strains have the ability to fix atmospheric dinitrogen into organic nitrogen-containing compounds, so displaying the simplest nutritional requirements of all microorganisms [4]. Cyanobacteria are also characterised by a great morphological diversity, unicellular as well as filamentous species being included with a cell volume ranging over more than five orders of magnitude [5]. Representatives of the group have been found, frequently in abundance, in most of the natural illuminated environments examined so far, both aquatic and terrestrial, including several types of extreme environments [5]. This widespread distribution reflects a large variety of species, covering a broad spectrum of physiological properties and tolerance to environmental stress [6]. Indeed, several cyanobacterial strains such as *chyoococcus sp.* (Figure 1(a)), *phormidium sp.* (Figure 1(b)) possess, outside their outer membrane, additional surface structures, mainly of a polysaccharidic nature, that comprise a wide variety of outermost investments differing in thickness, consistency, and appearance after staining. These structures, in spite of the rather arbitrary terminology sometimes used, can be referred to as three distinct types, namely, sheaths, capsules, and slimes. 

Over 300 nitrogen-containing secondary metabolites, represented by diverse structural types, have been reported from the prokaryotic marine cyanobacteria. A majority of these metabolites are biologically active and are products of either the nonribosomal polypeptide (NRP) or the mixed polyketide-NRP biosynthetic pathways. Biomolecules of the NRP and hybrid polyketide-NRP structural types are important subsets of natural products utilized as therapeutic agents.

These include the antibiotic vancomycin, the immunosuppressive agent cyclosporine, and the anticancer agent bleomycin [7]. Vancomycin is primarily effective against Gram-positive cocci. *Staphylococcus aureus *and *Staphylococcus epidermidis*, including both methicillin-susceptible (MSSA & MSSE) or resistant species (MRSA & MRSE), are usually sensitive to vancomycin. Vancomycin is also effective against the anaerobes, diphtheroids, and clostridium species, including *C. difficile*, whereas Bleomycin is a glycopeptide antibiotic produced by the bacterium *Streptomyces verticillus.* It works by causing breaks in DNA as anticancer drug. The drug is also used in the treatment of Hodgkin's lymphoma, squamous cell carcinomas, and testicular cancer, as well as in the treatment of plantar warts and as a means of effecting pleurodesis. The discovery of these unique classes of natural products from marine cyanobacteria represents an important source of novel microbial secondary metabolites, in addition to the actinomycetes and fungi, for drug discovery efforts. 

### 1.1. Anticancer Drugs from Marine Cyanobacteria

**Figure 2:**
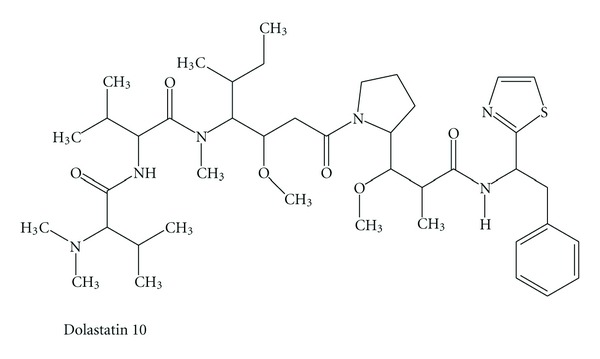
Structure of dolastatin 10.

**Figure 3:**
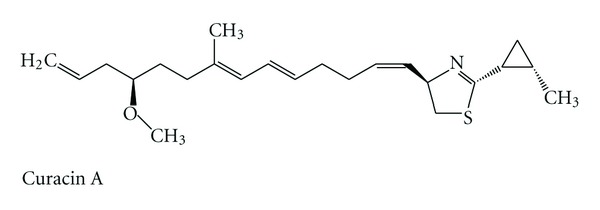
Structure of curacin A.

An increasing number of marine cyanobacterial compounds are found to target tubulin or actin filaments in eukaryotic cells, making them an attractive source of natural products as anticancer agents [8]. Prominent molecules such as the anti-microtubule agents, curacin A (Figure 3) and dolastatin 10 (Figure 2), have been in preclinical and/or clinical trials as potential anticancer drugs [9].

**Figure 4:**
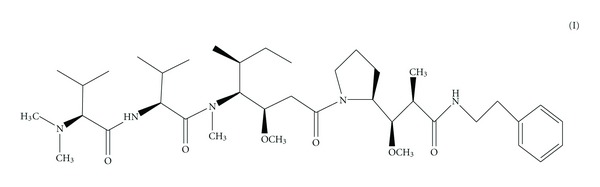
Structure of TZT-1027.

**Figure 5:**
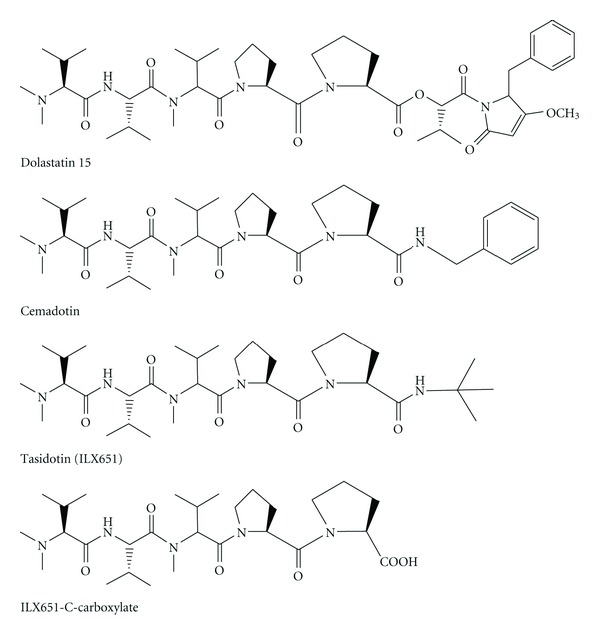
Structures of dolastatin-15, cemadotin, Tasidotin, and ILX651-C-carboxylate.

**Figure 6:**
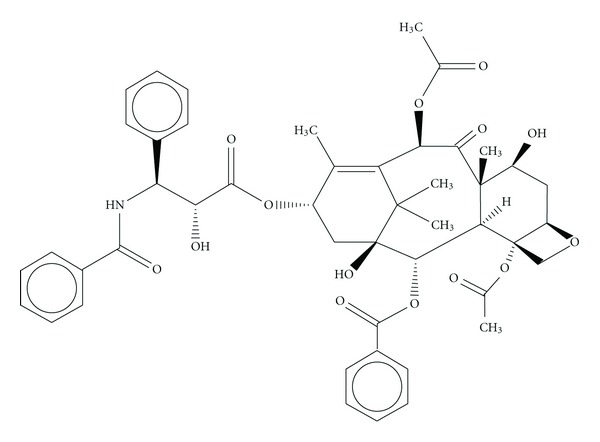
Structure of paclitaxel.

**Figure 7:**
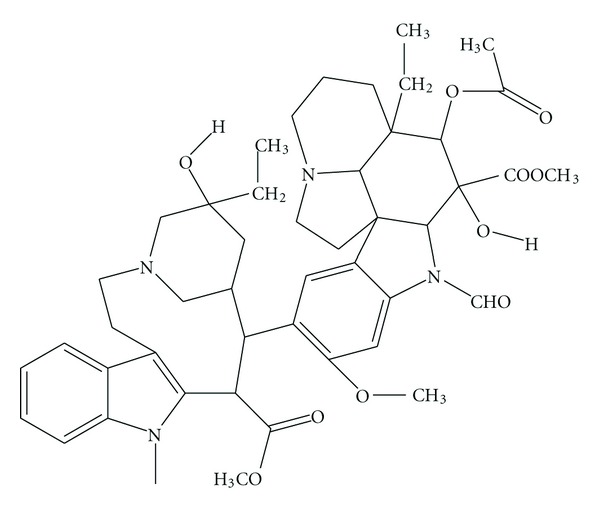
Structure of vincristine.

In addition, these molecules served as a drug leading to the development of synthetic analogues, for example, compound 4, TZT-1027 (Figure 4), ILX-651 (Figure 5), and LU-103793 (7), usually with improved pharmacologicaland pharmacokinetic properties for the treatment of different types of cancers. The antitumor activity of TZT-1027 (soblidotin) (Figure 4), a synthetic derivative of dolastatin 10 (Figure 2), was found to be superior to existing anticancer drugs, such as paclitaxel (Figure 6) and vincristine (Figure 7) and is currently undergoing Phase I testing for treating solid tumors [10].

The third generation dolastatin 15 analogue (Figure 5), ILX-651 (or tasidotin) (Figure 5), is another antitumor agent currently undergoing Phase II trials after its successful run in Phase I trials [11]. Pharmacological studies have also showed the mechanistic novelty of certain molecules, such as antillatoxin, in modifying the activity of Nav channels. These cyanobacterial toxins are source of valuable molecular tools in functional characterization of Nav channels as well as potential analgesics and neuroprotectants. 

The discovery of tiny, single-celled cyanobacteria as ubiquitous and abundant components of the marine microbiota has radically changed our view of the functioning and composition of marine ecosystems. It is now clear that the two genera *Prochlorococcus* and *Synechococcus* dominate the photoautotrophic picoplankton over vast tracts of the world's oceans where they occupy a key position at the base of the marine food web and contribute significantly to global primary productivity [12]. Cyanobacteria (blue-green algae) are worldwide in distribution, occurring in saline and nonsaline habitats of diverse ionic composition [13]. However, more emphasis is now being placed on the importance of various metabolic features as taxonomic markers in cyanobacteria. Recently it has been suggested that soluble organic compounds, accumulated as internal osmotica in response to salinity stress, may provide a major biochemical character which distinguishes marine and freshwater forms [5]. Thus glucosylglycerol has been considered to be “unique” to marine cyanobacteria [14], while sucrose has been reported to accumulate in response to osmotic stress in freshwater cyanobacteria [14]. 

### 1.2. Importance

Cyanobacteria in general and marine forms in particular are one of the richest sources of known and novel bioactive compounds including toxins with wide pharmaceutical applications [15]. Anti-HIV activity of marine cyanobacterial compounds from *Lyngbya lagerheimii * and *Phormidium tenue*. A massive programme of screening of extracts from the large culture collection of marine cyanobacteria for antiviral, antibacterial, antifungal, and immunomodulatory activities has resulted in recovery of a compound from marine *Oscillatoria laete*-virians BDU 20801 that shows anti-*Candida* activity. An immunopotentiating compound with male antifertility, without being toxic to other systems in a mice model, was found in the extracts of *Oscillatoria willei* BDU 130511 [16]. Medically important gamma linolenic acid (GLA) is relatively rich in cyanobacteria, namely, *Spirulina platensis* and *Arthrospira sp.* which is easily converted into arachidonic acid in the human body and arachidonic acid into prostaglandin E2 [17]. 

Prostaglandin E2 has lowering action on blood pressure and the contracting function of smooth muscle and thus plays an important role in lipid metabolism. The bioinformatic mining of cyanobacterial genomes has led to the discovery of novel cyanobactins. Heterologous expression of these gene clusters provided insights into the role of the genes participating in the biosynthesis of cyanobactins and facilitated the rational design of novel peptides.

### 1.3. Vitamins from Cyanobacteria

Some of the marine cyanobacteria appear to be potential sources for large-scale production of vitamins of commercial interest such as vitamins of the B complex group and vitamin E [18]. The carotenoids and phycobiliprotein pigments of cyanobacteria have commercial value as natural food colouring agents, as feed additives, as enhancers of the color of egg yolks, to improve the health and fertility of cattle, as drugs, and in the cosmetic industries. Some anti-HIV activity has been observed with the compounds extracted from *Lyngbya lagerhaimanii* and *Phormidium tenue * [18]. 

### 1.4. Biotechnology and Applications of Marine Cyanobacteria 

The unicellular cyanobacterium *Synechocystis sp*. PCC6803 was the third prokaryote and first photosynthetic organism whose genome was completely sequenced. It continues to be an important model organism. The smallest genomes have been found in *Prochlorococcus spp.* (1.7 Mb) and the largest in *Nostoc punctiforme* (9 Mb) [14]. Some cyanobacteria are sold as food, notably *Aphanizomenon flos-aquae* and *Arthrospira platensis* (Spirulina) [14]. Recent research has suggested the potential application of cyanobacteria to the generation of clean and green energy via converting sunlight directly into electricity. Currently efforts are underway to commercialize algae-based fuels such as diesel, gasoline, and jet fuel [19–24].

## 2. Secondary Metabolites from Marine Cyanobacteria 

### 2.1. Metabolic Themes and Building Blocks

There are currently some 300 marine cyanobacterial alkaloids. Of these, 128 marine cyanobacterial nitrogen-containing secondary metabolites. The majority of these biomolecules were isolated from the filamentous Order Nostocales, especially members belonging to the genera Lyngbya, Oscillatoria, and Symploca [20]. The locations of the collection sites were mainly from the tropics, including Papua New Guinea and the Pacific islands, in particular Guam and Palau. The predominant metabolic theme of nitrogen-containing marine cyanobacterial compounds is the occurrence of mixed polyketide-nonribosomal polypeptide structural types.

**Figure 8:**
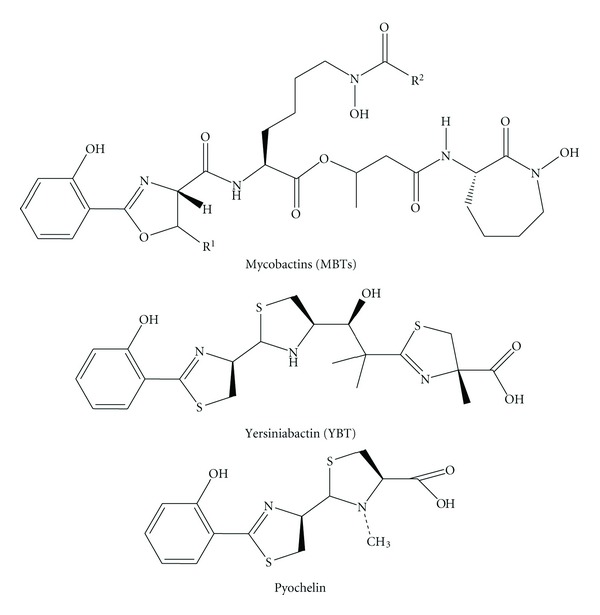
Structures of mycobactins, yersiniabactin, and pyochelin.

These are molecules containing acetate or propionate units as well as proteinogenic amino acids, forming as either linear or cyclic lipopeptides as found in mycobactins, Yersiniabactin and Pyochelin (Figure 8). The utilization of acetate-derived units in the construction of these hybrid compounds can be seen in several ways. Firstly, acetate-derived fatty acid chain can be coupled through amide bonds with a variety of functionalized amines in linear lipopeptides (e.g. malyngamides, S (41)–W (45)).

Further modifications on the fatty acid chain, such as methylation and halogenation, are common. Lipidation through amide bonds are also common in a number of oligopeptides, such as lyngbyabellin D and somamide. A Single acetate unit or multiple ketides can also be utilized to extend amino acids. For instance, a unit of acetate is used in the extension of a variety of amino acids, such as Ala, Phe, Pro, and Gly. The extension can either be linear or undergo cyclization to form common moieties, such as pyrrolinone ring system in the jamaicamides [21, 22].

Polyketide-derived moieties occurring as b-hydroxy or amino acid residues are source of nonproteinogenic units in the construction of lipopeptides, especially cyclic depsipeptides [22].

### 2.2. Nitrogen-Containing Lipids

Two new 2-alkypyridine alkaloids, phormidinines A (10) and B (11), were reported from an Okinawan collection of the marine cyanobacterium, *Phormidium sp*. [21, 22]. The structures and absolute stereochemistry of these compounds were determined based on 2D NMR spectra analysis and Mosher's method, respectively. A series of polychlorinated acetamides (12–16 and 19–29) and its dechlorinated derivatives (17 and 18) have been reported from *Microcoleus lyngbyaceus* and Lyngbyamajuscula/Schizothrix assemblage collected at Chuuk Island and Fiji, respectively [23]. A majority of these unique molecules are characterized by having terminal mono-, di-, or trichlorinated functional groups. Other marine cyanobacterial metabolites, for example, dysidenin-type compounds (e.g. 62) and barbamide (61), having terminal di- and trichloromethyl groups were shown to derive from chlorination of Leu, possibly via free radical mechanism. The biogenesis of the taveuniamides, isolated from Fijian Lyngbya majuscula/Schizothrix assemblage, has been proposed to occur either through the decarboxylation and methylation of an octaketide precursor or the C–C bond formation between the C-1 carboxyl carbon and C-2 of two tetraketide precursors [23].

## 3. Natural Products from Marine Cyanobacteria 

**Figure 9:**
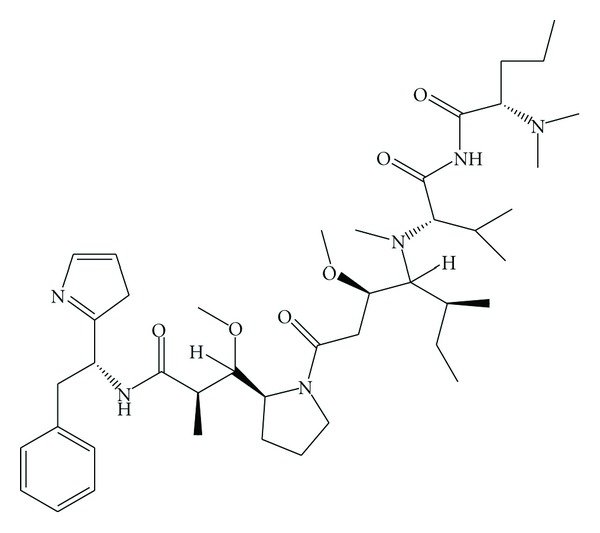
Structure of symplostatin 3.

**Figure 10:**
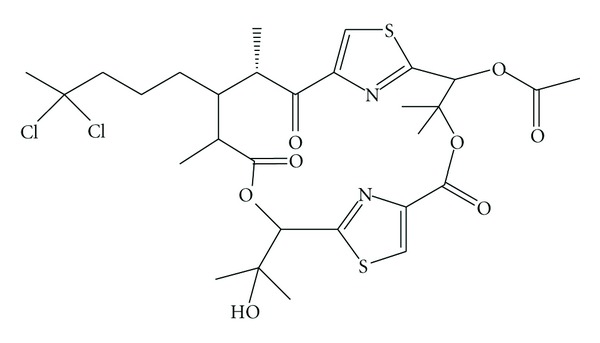
Structure of hectochlorin.

A number of highly potent cyanobacterial natural products have been uncovered as potential lead compounds for further drug development, especially in the area of anticancer agents. An increasing number of lipopeptides, such as symplostatin 3 (Figure 9), lyngbyastatin 3, hectochlorin (Figure 10), and lyngbyabellins (114–116 and 118–123), have been reported to target eukaryotic cytoskeletal macromolecules, such as actin and microtubule filaments [24]. 

These are attractive biological features for the development of potential anticancer drugs with specific cellular targets. Apratoxin A (126) is another potent cytotoxic compound worthy of further biological investigation as anticancer agent due to it mechanism of action in attenuating the FGF (fibroblast growth factor) signaling pathway [25]. Synthetic analogues based on the scaffolds of these cyanobacterial natural products can be developed for SAR studies as well as lead optimization for drug development [26].

## 4. Intramolecular Modulation of Serine Protease Inhibitor Activity in a Marine Cyanobacterium with Antifeedant Properties 

**Figure 11:**
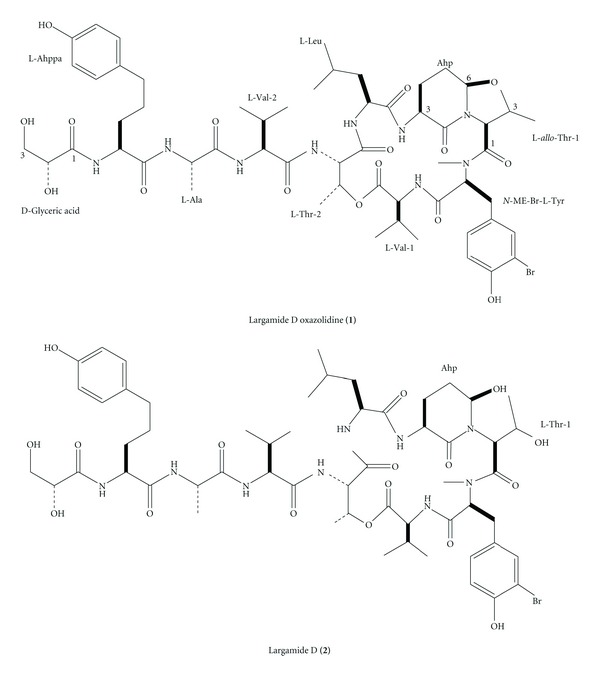
Structures of largamide D oxazolidihe (**1**), largamide D (**2**).

One prevalent class found in marine and freshwater *cyanobacteria *is comprised of protease inhibitors with a cyclic depsipeptide scaffold that contains a 3-amino-6-hydroxy-2-piperidone (Ahp) moiety as a key feature for inhibition of certain serine proteases [27]. Since many digestive enzymes such as trypsin and chymotrypsin are serine proteases and are inhibited by these compounds, these natural products could function as digestion inhibitors [28]. Serine protease inhibitors also cooccur with microcystins and are linked to an enhanced toxin activity or thought to upregulate biosynthetic genes [29]. The tropical sea urchin *Diadema antillarum*, which is a cyanobacterium, produces a wide array of serine protease inhibitors including lyngbyastatins 4–6 [30, 31], pompanopeptin A [32], and largamides D–G [33]. The antifeedant activity may be a reflection of the secondary metabolite content, known to be comprised of many serine protease inhibitors. Further chemical and NMR spectroscopic investigation led to isolate and structurally characterize a new serine protease inhibitor **1 **that is formally derived from an intramolecular condensation of largamide D (**2**) (Figure 11) [33]. The cyclization resulted in diminished activity, but to different extents against two serine proteases tested. This finding suggests that cyanobacteria can endogenously modulate the activity of their protease inhibitors.

## 5. Cyanobacteria: A Potential Source of New Biologically Active Substances

Cyanobacteria (blue-green algae) provide a potential source of biologically active secondary metabolites [34]. Investigations over the last decades have identified compounds with for instance cytotoxic, antifungal, antibacterial, or antiviral activity. Hydrophilic and lipophilic extracts of cyanobacterial strains, isolated from fresh and brackish water, and water blooms were investigated for their antibiotic activities against microorganisms both Gram negative and Gram positive. Most of the isolated substances belong to groups of polyketides, amides, alkaloids, and peptides [35].

The blue-green algae are among the oldest photoautotrophic organisms. Their cultivation without organic substrates can be an economical advantage over other microorganisms. In view of the growing resistance of bacteria to common antibiotics, the search for new antimicrobially active compounds has become increasingly important [36]. Screening programme was made which tested approximately fifty extracts from twelve different cyanobacterial strains and two water blooms against different bacteria and one yeast. The results of the study show the ability of cyanobacteria to produce compounds with antimicrobial effects [36].

### 5.1. Antimicrobial and Cytotoxic Assessment of Marine Cyanobacteria: Synechocystis and Synechococcus

Aqueous extracts and organic solvent extracts of isolated marine cyanobacteria strains were tested for antimicrobial activity against a fungus, Gram-positive and Gram-negative bacteria and for cytotoxic activity against primary rat hepatocytes and HL-60 cells [37]. Antimicrobial activity was based on the agar diffusion assay. Cytotoxic activity was measured by apoptotic cell death scored by cell surface evaluation and nuclear morphology [38]. A high percentage of apoptotic cells were observed for HL-60 cells when treated with cyanobacterial organic extracts [39]. Slight apoptotic effects were observed in primary rat hepatocytes when exposed to aqueous cyanobacterial extracts [40]. Marine Synechocystis and Synechococcus extracts induce apoptosis in eukaryotic cells and cause inhibition of Gram-positive bacteria. The different activity in different extracts suggests different compounds with different polarities [41].

## 6. Potential Commercial Development of Insecticides, Algaecides, and Herbicides from Cyanobacteria

Potential commercial development of cyanobacterial compounds for nonbiomedical applications, particularly including herbicides, algaecides, and insecticides poses a potentially important opportunity to utilize the biological activity of these compounds [26]. 

### 6.1. Insecticides

Fladmark et al.[42]. screened extracts from 76 isolates of cyanobacteria and found several of these isolates produced compounds that were larvicidal to *Aedes aegypti*. The greatest inhibition, however, was associated with presence of the hepatotoxic microcystins and the neurotoxic anatoxin-a. Humpage and Falconer[43] reported that, while investigating cyanobacteria as a biofertilizer, several strains were found to inhibit development of mosquito larvae, and subsequently showed that methanolic extracts from an isolate of *Westiellopsis sp. * were larvicidal to several species of mosquito, including representatives of *Aedes aegypti* (a vector for Dengue Fever), *Anopheles stephensi * (a vector for malaria), and *Culex tritaeniorhynchus* and *C. quinquefasciatus * (vectors of encephalitis). The use of genetically engineered cyanobacteria, specifically expressing the insecticidal proteins from *Bacillus thuringiensis* to control mosquito larvae [44, 45]. Likewise, cyanobacteria that produce naturally occurring larvicidal metabolites may eliminate the potential threats associated with release of transgenic organisms [44, 45].

## 7. Cyanobacterial Cyclopeptides as Lead Compounds to Novel Targeted Cancer Drugs

Cyanobacterial cyclopeptides, including microcystins and nodularins, are considered a health hazard to humans due to the possible toxic effects of high consumption [46]. From a pharmacological standpoint, microcystins are stable hydrophilic cyclic heptapeptides with a potential to cause cellular damage following uptake via organic anion transporting polypeptides (OATPs) [47]. Their intracellular biological effects involve inhibition of catalytic subunits of protein phosphatase 1 (PP1) and PP2, and glutathione depletion andgeneration of reactive oxygen species (ROS) [48]. Interestingly, certain OATPs are prominently expressed in cancers as compared to normal tissues, qualifying MC as potential candidates for cancer drug development. In targeted cancer therapy, cyanotoxins comprise a rich source of natural cytotoxic compounds with a potential to target cancers expressing specific uptake transporters [49]. Their structure offers opportunities for combinatorial engineering to enhance the therapeutic index and resolve organ-specific toxicity issues [50].

## 8. Conclusion

The fact that cyanobacteria are one of the richest sources of known and novel bioactive compounds including toxins with wide pharmaceutical applications is unquestionable. Many compounds from cyanobacteria are useful for welfare of mankind. Because of high discovery rate, research should be done to unfold other hidden aspects of marine cyanobacteria. An advantage of natural products research on marine cyanobacteria is the high discovery rate (>95%) of novel compounds as compared to other traditional microbial sources. This is due largely to the unexplored nature of this group of microalgae. One of the key areas to further tap these microalgae for new chemical entities is the collection of cyanobacterial strains from unexplored localities, especially from Africa and Asia. In addition to the procurement of marine cyanobacteria from unexplored locales, the amenability of field collected strains to laboratory culture is an important factor in the drug discovery process.

## References

[1] Castenholz RW, Waterbury JB (1989). Cyanobacteria. *Bergey's Manual of Systematic Bacteriology*.

[2] Smith AJ (1983). Modes of cyanobacterial carbon metabolism. *Annales de Microbiologie*.

[3] Cohen Y, Jrgensen BB, Revsbech NP, Paplawski R (1986). Adaptation to hydrogen sulfide of oxygenic and anoxygenic photosynthesis among cyanobacteria. *Applied and Environmental Microbiology*.

[4] Fay P (1992). Oxygen relations of nitrogen fixation in cyanobacteria. *Microbiological Reviews*.

[5] Whitton BA (1992). Diversity, ecology and taxonomy of the cyanobacteria. *Photosynthetic Prokaryotes*.

[6] Tandeau de Marsac N, Houmard J (1993). Adaptation of cyanobacteria to environmental stimuli: new steps towards molecular mechanisms. *FEMS Microbiology Reviews*.

[7] Schwarzer D, Finking R, Marahiel MA (2003). Nonribosomal peptides: from genes to products. *Natural Product Reports*.

[8] Jordan MA, Wilson L (1998). Microtubules and actin filaments: dynamic targets for cancer chemotherapy. *Current Opinion in Cell Biology*.

[9] Gerwick WH, Tan LT, Sitachitta N (2001). Nitrogen-containing metabolites from marine cyanobacteria. *The Alkaloids: Chemistry and Biology*.

[10] Watanabe J, Minami M, Kobayashi M (2006). Antitumor activity of TZT-1027 (soblidotin). *Anticancer Research*.

[11] Mita AC, Hammond LA, Bonate PL (2006). Phase I and pharmacokinetic study of tasidotin hydrochloride (ILX651), a third-generation dolastatin-15 analogue, administered weekly for 3 weeks every 28 days in patients with advanced solid tumors. *Clinical Cancer Research*.

[12] Blumenthal KM, Seibert AL (2003). Voltage-gated sodium channel toxins: poisons, probes, and future promise. *Cell Biochemistry and Biophysics*.

[13] Reed RH, Borowitzka LJ, Mackay MA (1986). Organic solute accumulation in osmotically stressed cyanobacteria. *FEMS Microbiology Reviews*.

[14] Dufresne A, Martin O, David JS (2008). Unraveling the genomic mosaic of a ubiquitous genus of marine cyanobacteria. *Genome Biology*.

[15] Raghavan C, Kadalmani B, Thirunalasundari T, Subramanian G, Akbarsha MA (2002). *Biological and Comparative Endocrinology*.

[16] Deth SK (1999). *Antimicrobial compounds from marine cyanobacteria with special reference to the bioactivity of a purified compound from *Oscillatoria laete-virens* BDU 20801*.

[17] Euler US, Eliassen R (1967). *Prostaglandins*.

[18] Schaeffer DJ, Krylov VS (2001). *Anti-HIV Activity of Extracts and Compounds from Algae and Cyanobacteria Department of Veterinary Biosciences*.

[19] Kaneko T, Tanaka A, Sato S (1995). Sequence analysis of the genome of the unicellular cyanobacterium *Synechocystis* sp. strain PCC6803. I. Sequence features in the 1 Mb region from map positions 64% to 92% of the genome. *DNA Research*.

[20] Gerwick WH, Tan LT, Sitachitta N (2001). Nitrogen-containing metabolites from marine cyanobacteria. *Alkaloids: Chemistry and Biology*.

[21] Teruya T, Kobayashi K, Suenaga K, Kigoshi H (2005). Phormidinines A and B, novel 2-alkylpyridine alkaloids from the cyanobacterium *Phormidium* sp. *Tetrahedron Letters*.

[22] Orsini MA, Pannell LK, Erickson KL (2001). Polychlorinated acetamides from the cyanobacterium *Microcoleus lyngbyaceus*. *Journal of Natural Products*.

[23] Meeks JC (2005). An overview of the genome of *Nostoc punctiforme*, a multicellular, symbiotic cyanobacterium. *Current Science*.

[24] Spolaore P, Joannis-Cassan C, Duran E, Isambert A (2006). Commercial applications of microalgae. *Journal of Bioscience and Bioengineering*.

[25] Lehane L, Lewis RJ (2000). Ciguatera: recent advances but the risk remains. *International Journal of Food Microbiology*.

[26] Shimizu Y (2003). Microalgal metabolites. *Current Opinion in Microbiology*.

[27] Gunasekera SP, Miller MW, Kwan JC, Luesch H, Paul VJ (2010). Molassamide, a depsipeptide serine protease inhibitor from the marine cyanobacterium *Dichothrix utahensis*. *Journal of Natural Products*.

[28] Kwan JC, Taori K, Paul VJ, Luesch H (2009). Lyngbyastatins 8-10, elastase inhibitors with cyclic depsipeptide scaffolds isolated from the marine cyanobacterium *Lyngbya semiplena*. *Marine Drugs*.

[29] Radau G (2005). Cyanopeptides: a new and nearly inexhaustible natural resource for the design and structure-activity relationship studies of the new inhibitors of trypsin-like serine proteases. *Current Enzyme Inhibition*.

[30] Taori K, Matthew S, Ross C, James RR, Paul VJ, Luesch H (2007). Lyngbyastatins 5-7, potent elastase inhibitors from Floridian marine cyanobacteria, *Lyngbya* spp. *Journal of Natural Products*.

[31] Matthew S, Ross C, Paul VJ, Luesch H (2008). Pompanopeptins A and B, new cyclic peptides from the marine cyanobacterium *Lyngbya confervoides*. *Tetrahedron*.

[32] Plaza A, Bewley CA (2006). Largamides A-H, unusual cyclic peptides from the marine cyanobacterium *Oscillatoria* sp. *Journal of Organic Chemistry*.

[33] Gunasekera SP, Ritson-Williams R, Paul VJ (2008). Carriebowmide, a new cyclodepsipeptide from the marine cyanobacterium *Lyngbya polychroa*. *Journal of Natural Products*.

[34] Cruz-Rivera E, Paul VJ (2007). Chemical deterrence of a cyanobacterial metabolite against generalized and specialized grazers. *Journal of Chemical Ecology*.

[35] Thacker RW, Nagle DG, Paul VJ (1997). Effects of repeated exposures to marine cyanobacterial secondary metabolites on feeding by juvenile rabbitfish and parrotfish. *Marine Ecology Progress Series*.

[36] Paul VJ, Thacker RW, Banks K, Golubic S (2005). Benthic cyanobacterial bloom impacts the reefs of South Florida (Broward County, USA). *Coral Reefs*.

[37] Tan LT, Sitachitta N, Gerwick WH (2003). The guineamides, novel cyclic depsipeptides from a Papua New Guinea collection of the marine cyanobacterium **Lyngbya majuscula**. *Journal of Natural Products*.

[38] Bunyajetpong S, Yoshida WY, Sitachitta N, Kaya K (2006). Trungapeptins A-C, cyclodepsipeptides from the marine cyanobacterium *Lyngbya majuscula*. *Journal of Natural Products*.

[39] Tan LT, Márquez BL, Gerwick WH (2002). Lyngbouilloside, a novel glycosidic macrolide from the marine cyanobacterium *Lyngbya bouillonii*. *Journal of Natural Products*.

[40] Sponga F, Cavaletti L, Lazzarini A (1999). Biodiversity and potentials of marine-derived microorganisms. *Journal of Biotechnology*.

[41] Mayer AMS, Gustafson KR (2003). Marine pharmacology in 2000: antitumor and cytotoxic compounds. *International Journal of Cancer*.

[42] Fladmark KE, Serres MH, Larsen NL, Yasumoto T, Aune T (1998). Sensitive detection of apoptogenic toxins in suspension cultures of rat and salmon hepatocytes. *Toxicon*.

[43] Humpage AR, Falconer IR (1999). Microcystin-LR and liver tumor promotion: effects on cytokinesis, ploidy, and apoptosis in cultured hepatocytes. *Environmental Toxicology*.

[44] Angsuthanasombat C, Panyim S (1989). Biosynthesis of 130-kilodalton mosquito larvicide in the cyanobacterium *Agmenellum quadruplicatum* PR-6. *Applied and Environmental Microbiology*.

[45] Murphy RC, Stevens SE (1992). Cloning and expression of the cryIVD gene of Bacillus thuringiensis subsp. israelensis in the cyanobacterium *Agmenellum quadruplicatum* PR-6 and its resulting larvicidal activity. *Applied and Environmental Microbiology*.

[46] Soni B, Trivedi U, Madamwar D (2008). A novel method of single step hydrophobic interaction chromatography for the purification of phycocyanin from *Phormidium fragile* and its characterization for antioxidant property. *Bioresource Technology*.

[47] Francis G (1878). Poisonous Australian lake. *Nature*.

[48] Gupta N, Pant SC, Vijayaraghavan R, Rao PVL (2003). Comparative toxicity evaluation of cyanobacterial cyclic peptide toxin microcystin variants (LR, RR, YR) in mice. *Toxicology*.

[49] Falconer IR, Humpage AR (2005). Health risk assessment of cyanobacterial (blue-green algal) toxins in drinking water. *International Journal of Environmental Research and Public Health*.

[50] Vareli K, Briasoulis E, Pilidis G, Sainis I (2009). Molecular confirmation of Planktothrix rubescens as the cause of intense, microcystin-Synthesizing cyanobacterial bloom in Lake Ziros, Greece. *Harmful Algae*.

